# *Echinococcus vogeli* Infection in a Hunter, French Guiana

**DOI:** 10.3201/eid1512.090940

**Published:** 2009-12

**Authors:** Jenny Knapp, Mircea Chirica, Christine Simonnet, Frederic Grenouillet, Jean-Mathieu Bart, Yasuhito Sako, Sonoyo Itoh, Minoru Nakao, Akira Ito, Laurence Millon

**Affiliations:** Université de Franche-Comté, Besançon, France (J. Knapp, F. Grenouillet, J.-M. Bart, L. Millon); Asahikawa Medical College, Asahikawa, Hokkaido, Japan (J. Knapp, Y. Sako, S. Itoh, M. Nakao, A. Ito); Saint-Louis Hospital, Paris, France (M. Chirica); Pasteur Institute of French Guiana, Cayenne, French Guiana (C. Simonnet); World Health Organization Collaborating Center for Prevention and Treatment of Human Echinococcosis, Besançon (J. Knapp, F. Grenouillet, L. Millon); 1 These authors contributed equally to this article.

**Keywords:** zoonosis, helminthic infection, parasites, Echinococcus vogeli, French Guiana, dispatch

## Abstract

*Echinococcus vogeli* infection in a hunter from the rain forest of French Guiana was confirmed by imaging and mitochondrial DNA sequence analysis. Serologic examination showed typical patterns for both alveolar and cystic echinococcosis. Polycystic echinococcis caused by *E. vogeli* may be an emerging parasitic disease in Central and South America.

Echinococcosis is one of the most lethal helminthic zoonoses worldwide. The 4 species of the genus *Echinococcus* are *E. granulosus* sensu lato, now including 5 independent species (*1*,*2*), which causes cystic echinococcosis (CE); *E. multilocularis,* which causes alveolar echinococcosis; *E. vogeli,* which causes polycystic echinococcosis (PE); and *E. oligarthrus,* which causes the recently described unicystic echinococcosis (*3*–*6*). Among these species, *E. oligarthrus* and *E. vogeli* are neotropical species localized exclusively in Central and South America (*5*,*6*). Only 3 cases of *E. oligarthrus* infection have been reported in the literature (1 from Brazil, 1 from Venezuela, and 1 from Surinam); 168 *E. vogeli* cases have been reported in 12 countries in Central and South America. To date, there have been no reports of neotropical echinococcosis in Bolivia, Paraguay, Guyana, or French Guiana (*5*,*6*). *E. granulosus* occurs sympatrically in South America, whereas *E. multilocularis* does not occur there at all (*3*,*5*). As both *E. vogeli* and *E. oligarthrus* have primarily sylvatic life cycles and the diagnosis is usually based on histopathologic examination of resected lesions, the number of human cases might be underestimated because of the small number of patients who receive surgical treatment (*5**,**6*)*.* We report a case of human infection from French Guiana caused by *E. vogeli*.

## The Study

**Figure 1:**
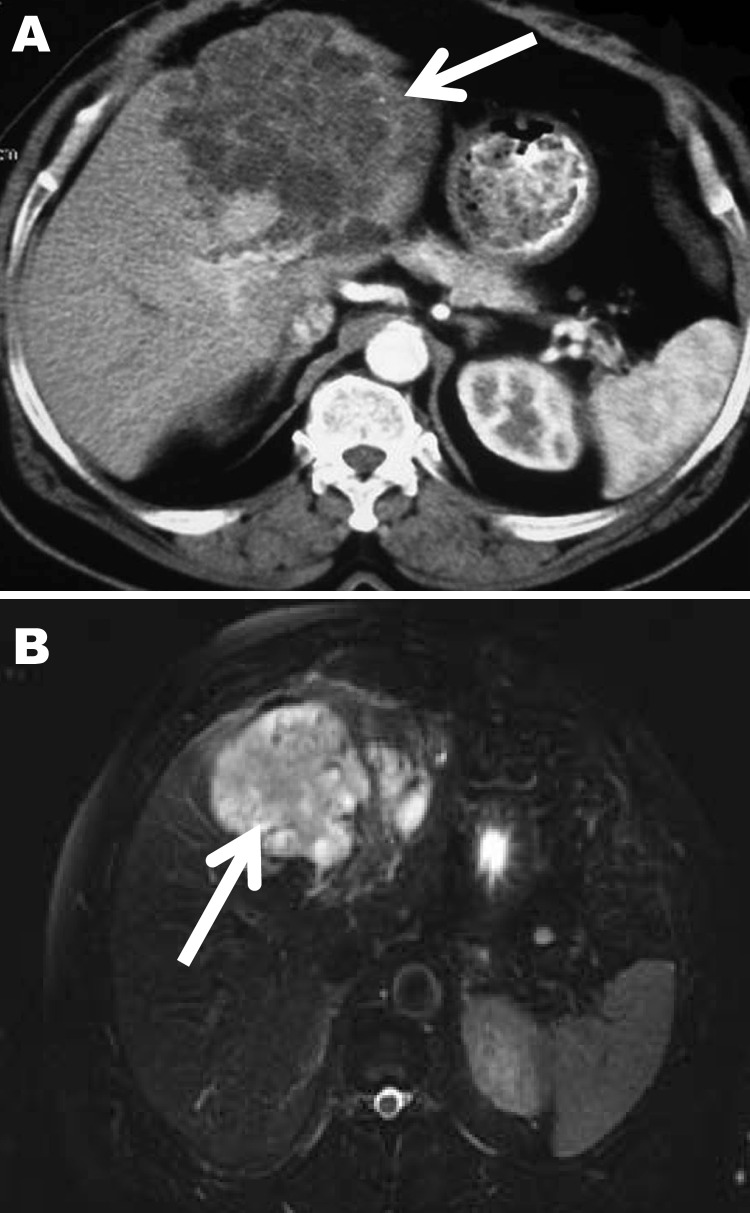
Computed tomography (A) and magnetic resonance (B) images of the liver of a 72-year-old man from French Guiana with polycystic echinococcosis affecting the left side of the liver. White arrows indicate the multicystic liver lesion.

**Figure 2:**
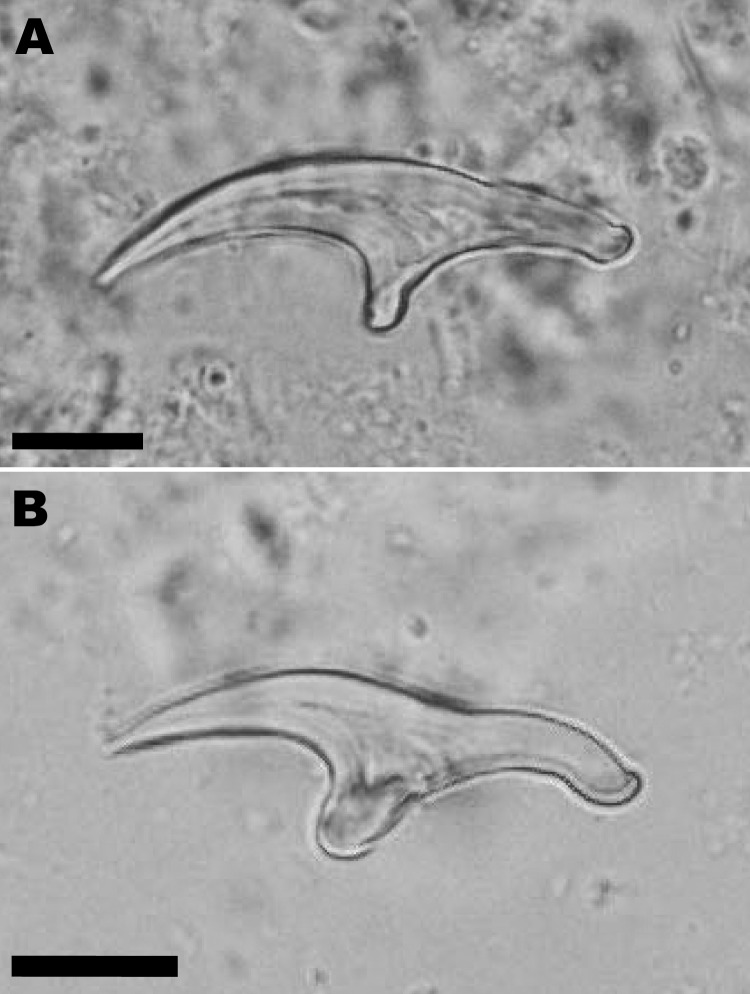
Large (A) and small (B) hooks from *Echinococcus vogeli* protoscoleces in the liver lesion of a 72-year-old man from French Guiana. Scale bars = 10 μm.

In April 2006, a 72-year-old man was admitted to a local hospital in Cayenne, French Guiana, for abdominal pain and a palpable epigastric mass. The patient hunted jaguars in the rain forest of French Guiana and owned dogs. He had no history of travel outside French Guiana. An exploratory laparotomy performed in June 2006 showed a hard, whitish liver tumor, deemed unresectable. Histopathologic examination of a biopsied sample of the tumor showed multilocular cysts. Albendazole treatment was started immediately after surgery. In January 2007, the patient was referred to the Department of General, Endocrine, and Digestive Surgery at the Saint-Louis Hospital, Paris, France. Computed tomography showed a multilocular hypoattenuating cystic mass in the left side of the liver, infiltrating the left glissonian pedicle up to the hepatic hilum (Figure 1, Panel A). Magnetic resonance imaging showed a well-defined, thin-walled, multilocular cystic mass (11 × 10 × 12 cm) involving segments II, III, and IV of the liver (Figure 1, Panel B) and multiple intraperitoneal cysts. The cysts appeared markedly hyperintense on T2-weighted images, hypo-intense on T1-weighted images, and showed slight enhancement of the septa after gadolinium injection. The patient underwent a left hepatectomy and resection of intraperitoneal cysts. Analysis of the operative specimen showed multiple large and small parasite hooks, with mean lengths of 32.7 µm ± 1.6 µm and 42.7 µm ± 0.7 µm, respectively (Figure 2).

Two serum samples from the patient (one obtained in May 2006, the other in December 2006) were analyzed by several immunologic techniques; all indicated infection with *Echinococcus* spp. Commercial ELISAs using *E. granulosus* antigens (Bordier Affinity Products, Lausanne, Switzerland, and Biotrin International, Antony, France) and the *E. multilocularis*-specific Em2^plus^-ELISA (Bordier Affinity Products, Switzerland) were both strongly positive. Confirmative Western blots (LD Bio Products, Lyon, France) showed a shadow at 16–18 kDa, which is characteristic for *E. granulosus* infection, on both samples. Additional Western blots carried out at Asahikawa Medical College (Asahikawa, Japan) using recombinant antigens (RecAgB8/1, more specific for *E. granulosus*, and RecEm18, more specific for *E. multilocularis*; *7*,*8*) also showed strong responses. On the basis of serologic patterns obtained with recombinant antigens, with no knowledge of the patient’s clinical background, travel history, or images of the lesion, *E. granulosus* infection with many multiple cysts, advanced *E. multilocularis*, or advanced *E. vogeli* infection was suspected (*7*,*9*).

Molecular identification was carried out with reference to the GenBank database by using a highly polymorphic DNA target (*10*). DNA from the liver lesion was extracted by using the High Pure PCR Preparation Kit (Roche, Mannheim, Germany). A part of the *cox1* mitochondrial gene was sequenced by using the *E. vogeli*–specific primer set (*cox1*_F: 5′-TTAATTTTGCCTGGGTTTGG-3′ and *cox1*_R: 5′-ACGACCCATATGATCCCAAA-3′). A sequence of 492 bp was obtained with the ABI 310 sequencer (Applied Biosystems, Foster City, CA, USA) and was compared with *Echinococcus* spp. sequences published in the GenBank database (Appendix Table). The sequences were aligned by using BioEdit 7.0.9.0 (*11*), and sequence identity matrix was generated based on the percentage of base pairs in common between species. The *cox1* sequence was found to be 100% identical to *E. vogeli* species originating from Colombia (GenBank accession no. AB208546; *2*) and was clearly distinguishable from all other *Echinococcus* species (Appendix Table).

On the basis of imaging showing numerous multiple cysts, serologic examination showing typical patterns for both alveolar and cystic echinococcosis, and the life history of the patient, the diagnosis of polycystic echinococcosis caused by *E. vogeli* could have been made before surgical intervention (*5*,*7*,*9*). The immunoblot showing a strong antibody response to recombinant AgB suggested a large volume of cyst fluid. Therefore, the immunoblot showing strong responses to both recombinant Em18 and AgB may be a typical pattern for advanced *E. vogeli* infection (data not shown). Because few studies using serologic analysis on human *E. vogeli* cases have been published, it would be useful to study antibody responses using recombinant antigens with large numbers of such patients and to compare the results with patterns observed with alveolar and cystic echinococcosis (*7*,*9*).

After surgery, identification of parasite hooks was carried out. The hooks showed the characteristic shape of *E. vogeli* and thus differed from *E. granulosus* (mean lengths of large hooks 25.9–35 μm, and small hooks 22.6–27.8 µm) and *E. oligarthrus* (30.5–33.4 µm and 25.4–27.3 µm, respectively) (*12*,*13*). *E. granulosus* and *E. oligarthrus* also occur in South America. The presence of hooks indicated that the parasite lesion was fertile in our patient, as shown in ≈50% of cases (*5*). Based on mitochondrial DNA analysis, the parasite identification was confirmed as *E. vogeli*. Further molecular studies on the haplotypes of this species may give information concerning the genetic diversity and circulation of the parasite in South America (*14*).

Albendazole has been used for medical management of alveolar and advanced cystic echinococcosis (*3*). Several instances of its efficacy on polycystic echinococcosis have been reported, but given the primacy of surgical management of these infections, albendazole will probably remain an additional treatment (*5*).

Neotropical echinococcosis cases are rare compared with alveolar and cystic echinococcosis (*5*). This rarity is probably because of the sylvatic life cycle of these species. However, because domestic dogs have been introduced to areas where *E. vogeli* is present in its natural cycle, the potential for transmission of the parasites from dogs to humans by close contact exists. The at-risk population mainly lives in rural areas and has limited access to medical services, which strongly suggests that many infected persons cannot receive adequate treatment for this underestimated disease.

## Conclusions

We report an autochthonous case of *E. vogeli* infection documented in French Guiana. Further investigations are needed to improve the serologic diagnosis of this infection and to define its typical serologic pattern compared with echinococcosis. Healthcare providers need to be alert to the existence of neotropical echinococcosis and should consider the possibility of its emergence in Central and South America. Although rare, this disease is still lethal in untreated cases

## Supplementary Material

Appendix Table Sequence identity matrix among Echinococcus spp. Cox1 referenced sequences (a 492-bp part of the cox1 gene)*
