# Supplementary material for: Echinococcus vogeli Infection in a Hunter, French Guiana
Source: Emerg Infect Dis. 2009 Dec;15(12):2029–31. doi: 10.3201/eid1512.090940 (PMC3044547; doi:10.3201/eid1512.090940)
Supplement: Appendix Table — Sequence identity matrix among Echinococcus spp. Cox1 referenced sequences (a 492-bp part of the cox1 gene)* [file 09-0940_appT-s1.pdf]

Appendix Table. Sequence identity matrix among *Echinococcus* spp. Cox1 referenced sequences (a 492-bp part of the *cox1* gene)*

Cox1 referenced sequences	GenBank accession no.	*E. multilocularis*	*E. shiquicus*	*E. granulosus*	*E. equinus*	*E. ortleppi*	*E. canadensis* G6	*E. canadensis* G7	*E. canadensis* G8	*E. oligarthrus*	*E. vogeli*
E. multilocularis	AB018440										
E. shiquicus	AB208064	92.4									
E. granulosus	AF297617	91.4	91.6								
E. equinus	AF346403	92.4	93.2	92.8							
E. ortleppi	AB235846	92.4	91.2	92.0	92.6						
E. canadensis G6	AB208063	91.0	90.0	91.8	91.0	94.9					
E. canadensis G7	AB235847	91.0	90.0	91.8	91.0	94.9	100				
E. canadensis G8	AB235848	92.0	89.6	90.6	91.8	94.7	95.5	95.5			
E. oligarthrus	AB208545	91.8	91.6	90.0	91.4	91.4	89.8	89.8	90.0		
E. vogeli	AB208546	91.8	91.6	91.4	93.6	91.4	90.0	9.00	90.4	91.4	
DNA of samples of parasite from patient		91.8	91.6	91.4	93.6	91.4	90.0	90.0	90.4	91.4	100

*Identity is provided as the percentage of nucleotide base differences.
